# To Prevent Death by Chloroform

**Published:** 1870-01

**Authors:** 


To Prevent Death by Chloroform.—Experiments on inferior animals show that they may be restored from apparent death from chloroform by the continuous galvanic current, the negative pole being put in the mouth and the positive pole in the rectum. In some cases the animal was left for two minutes in a state of apparent death and then restored. —*Physician and Pharmaceutist.*

